# Developing a novel positronium biomarker for cardiac myxoma imaging

**DOI:** 10.1186/s40658-023-00543-w

**Published:** 2023-03-24

**Authors:** Paweł Moskal, Ewelina Kubicz, Grzegorz Grudzień, Eryk Czerwiński, Kamil Dulski, Bartosz Leszczyński, Szymon Niedźwiecki, Ewa Ł. Stępień

**Affiliations:** 1 Department of Experimental Particle Physics and Applications, Marian Smoluchowski Institute of Physics, Faculty of Physics, Astronomy and Applied Computer Science, Jagiellonian University, Łojasiewicza 11, 30-348 Kraków, Poland; 2 Total-Body Jagiellonian-PET Laboratory, Jagiellonian University, Kraków, Poland; 3 Center for Theranostics, Jagiellonian University, Kraków, Poland; 4 Department of Medical Physics, Marian Smoluchowski Institute of Physics, Faculty of Physics, Astronomy and Applied Computer Science, Jagiellonian University, Łojasiewicza 11, 30-348 Kraków, Poland; 5 Department of Cardiovascular Surgery and Transplantology, John Paul II Hospital, Kraków, Poland; 6 Department of Cardiovascular Surgery and Transplantology, Jagiellonian University Medical College, Kraków, Poland

**Keywords:** Biomarker, Myxoma, PET, Positronium

## Abstract

**Purpose:**

Cardiac myxoma (CM), the most common cardiac tumor in adults, accounts for 50–75% of benign cardiac tumors. The diagnosis of CM is often elusive, especially in young stroke survivors and transthoracic echocardiography (TTE) is the initial technique for the differential diagnostics of CM. Less invasive cardiac computed tomography (CT) and magnetic resonance imaging (MRI) are not available for the majority of cardiac patients. Here, a robust imaging approach, ortho-Positronium (o-Ps) imaging, is presented to determine cardiac myxoma extracted from patients undergoing urgent cardiac surgery due to unexpected atrial masses. We aimed to assess if the o-Ps atom, produced copiously in intramolecular voids during the PET imaging, serves as a biomarker for CM diagnosing.

**Methods:**

Six perioperative CM and normal (adipose) tissue samples from patients, with primary diagnosis confirmed by the histopathology examination, were examined using positron annihilation lifetime spectroscopy (PALS) and micro-CT. Additionally, cell cultures and confocal microscopy techniques were used to picture cell morphology and origin.

**Results:**

We observed significant shortening in the mean o-Ps lifetime in tumor with compare to normal tissues: an average value of 1.92(02) ns and 2.72(05) ns for CM and the adipose tissue, respectively. Microscopic differences between tumor samples, confirmed in histopathology examination and micro-CT, did not influenced the major positronium imaging results.

**Conclusions:**

Our findings, combined with o-Ps lifetime analysis, revealed the novel emerging positronium imaging marker (o-PS) for cardiovascular imaging. This method opens the new perspective to facilitate the quantitative in vivo assessment of intracardiac masses on a molecular (nanoscale) level.

## Introduction

The advent of total-body positron emission tomography (TB-PET) has opened a new paradigm for personalized care in precision medicine, enabling simultaneous kinetic and parametric molecular imaging of all tissues in the human body [1–10]. The long axial field of view (covering the complete human body) and high sensitivity of TB-PET (40 times higher than standard PET scanners) enable the dynamic total-body scan at one bed position. This in turn facilitates quantitative diagnostic analyses based on parametric images, for example, the influx rate of radiopharmaceutical uptake, in addition to the conventional semiquantitative standardized uptake value (SUV)-based diagnostics [11, 12]. Moreover, the high sensitivity of TB-PET has developed new prospects for introducing in medicine novel diagnostic parameters based on positronium atoms, which are copiously produced in the human body during regular PET imaging [1–4, 13–15].

**Fig. 1:**
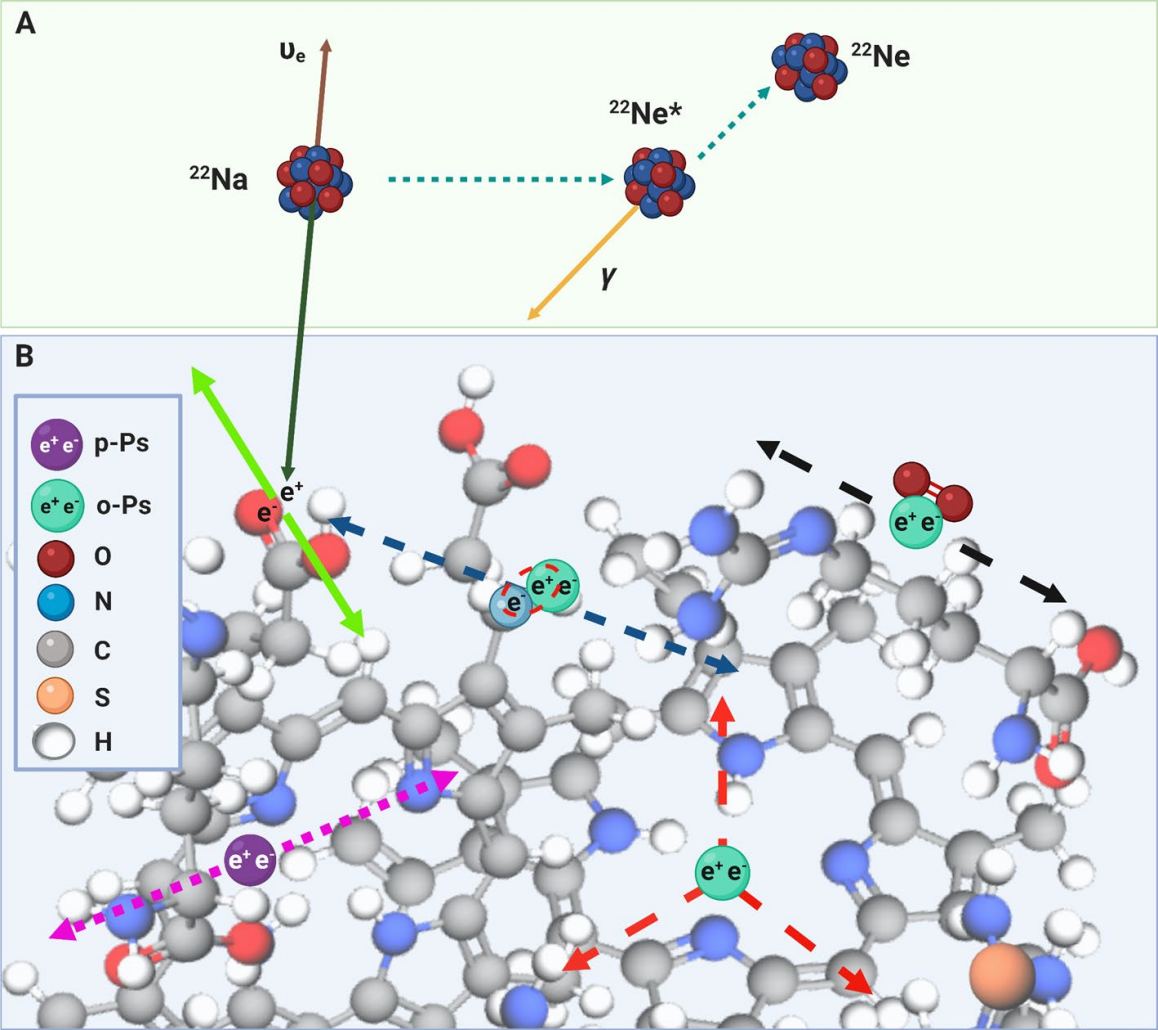
A pictorial illustration of the basic processes leading to the formation and decay of positronium in the intramolecular voids of a hemoglobin molecule. We have used a sodium ^**22**^Na isotope as an emitter of positrons (e +), considering the role of sodium fluoride in cardiovascular imaging [34, 37]. **a** ^**22**^Na radionuclide decays emitting a neutrino (brown arrow) and a positron (dark green arrow) (e +), and turns into an excited ^**22**^Ne* nucleus. It de-excites almost instantly (on an average in 3 ps) by the emission of the prompt photon (yellow arrow). **b** The positron thermalizes at a distance of about 1 mm [38], and annihilates into photons with one of the electrons (e^−^) in the surrounding molecules. Positron–electron annihilation in the tissue undergoes direct annihilation into two photons (solid light green arrows) in roughly 60% of the cases. However, it proceeds via the formation of positronium atom in 40% of the cases. The latter may be trapped in the tissue in the intramolecular voids [39]. Positronium atom can be created in two forms: (i) short-lived (125 ps) para-Positronium (p-Ps indicated in purple), which decays into two photons (dotted pink arrows) or (ii) long-lived (142 ns) ortho-Positronium (o-Ps indicated in mint), which decays into three photons (dashed red arrows). In the tissue, o-Ps predominantly annihilates either through an interaction with an electron (e^−^) from the surrounding molecule via pick-off process (dashed blue arrows) or through the conversion to p-Ps via an interaction with oxygen molecules, which subsequently decays into two photons (dashed black arrows) [16, 40]. These processes decrease the o-Ps lifetime, which becomes strongly dependent on the size of intramolecular voids and the concentration of bioactive molecules

Positronium is an exotic atom created from an electron (present in biomolecules) and a positron emitted by a radionuclide while being a part of a radiopharmaceutical. Figure 1 outlines the basic processes that lead to the formation and decay of positronium in the hemoglobin molecule. In PET, as much as 40% of positron–electron annihilations occurs through the production of positronium atoms in the inter and intramolecular spaces (voids) inside a patient’s body [1–4, 13–15]. The properties of these positronium atoms, such as lifetime and decay rate, depend on the size of molecular voids as well as the concentrations of biomolecules in tissues and biofluids. Moreover, they could provide information about the disease status or tumor microenvironment for discrimination a hypoxic region [4, 16]. In previous studies, we hypothesized that positronium imaging capabilities combined with TB-PET systems may lead to significant improvements in diagnostic and prognostic assessments [1–4, 13–15]. Total body approach will bring an advantage in the early diagnosis and treatment of various diseases [10], including oncological [17], cardiovascular [18], systemic and neurological [19, 20], thus enabling the simultaneous detection of pathologies on a molecular level, before they lead to functional or structural abnormalities [1, 3].

In this article, we demonstrate the strength of novel positronium diagnostic parameters for the in vivo assessment of benign cardiac tumors. We measure and analyze the mean positronium lifetime in a cardiac neoplasm (myxoma) and compare it with data obtained from normal mediastinal adipose tissues.

Cardiac myxoma (CM) was first described by Polish pathologist W. L. Brodowski in 1867 as a walnut size jelly-like tumor dissected from the left atrium of a 40-year-old woman who died of parenchymal nephritis [21]. The first description of five left ancient ventricular polypus specimens was summarized by T. W. King in 1845 [22]. CM is the most common cardiac tumor in adults and accounts for approximately 50–75% and > 50% of benign and primary cardiac neoplasms, respectively [23–28]. Left atrial CM is twice most common in women, compared to men [29]. Considering its left-atrial localization, CM is an origin of emboli to the vascular tree, particularly to the central nervous system [29]. It results in stroke or transient ischemic attack, particularly in young adults [30, 31]. Therefore, CM is a life-treating disease that increases the risk of severe systemic and cardiac symptoms, perioperative morbidity, and mortality [29]. The etiology of CM is poorly understood. Nevertheless, researchers have confirmed the presence of cardiomyocytes and mesenchymal progenitor factors (Nkx2.5/Csx, c-kit) in CM cells, thus revealing the stem cell origin of myxoma-initiating cells [32, 33].

The diagnosis of CM is often elusive, especially in young stroke survivors [31]. This can be attributed to their non-specific neurological symptoms. Transthoracic echocardiography (TTE) is the initial technique for the differential diagnostics of CM [23, 28, 29]. It provides additional information about the hemodynamic parameters and enables the characterization of intracardiac masses, in terms of their size and morphology. However, TTE fails to distinguish between thrombi, vegetation, or neoplasms [23]. In practice, cardiac computed tomography (CT) and magnetic resonance imaging (MRI) are less available for the majority of patients. Furthermore, the preoperative imaging diagnosis appears to be discordant with histopathologic findings in one-fifth and more than half cardiac tumors and malignant lesions, respectively. This in turn limits CM recognition.

PET is used as standard in cardiovascular imaging, for example, for semiquantitative myocardial perfusion evaluation [34, 35]. For research an assessment of atherosclerosis [18, 36] or the diagnosis and risk stratification in patients with known or suspected coronary artery disease [34, 37]. Nevertheless, standard PET does not reveal the tissue histology and fails to determine if the organic cause of the detected disorder is cancer origin, inflammation, or intracardiac thrombus.

Herein, we first present the results indicating that the in vivo imaging of positronium properties, available because of TB-PET scanners [1–4, 13–15], would facilitate the quantitative in vivo assessment of the intracardiac masses. Our findings would shift imaging diagnostics toward a new direction of personalized medicine.

## Materials and methods

### Study group characteristics

We analyzed and compared non-fixed myxoma samples with adipose tissues obtained from the patients. Each patient completed a screening interview, laboratory test (blood counts and metabolic panel), electrocardiogram, and urine toxicology. In all cases, the patients were diagnosed by TTE. Therefore, a histopathological examination of the specimen postoperatively confirmed the structure type. Table 1 summarizes information on the patient's health, type of tumor, and other demographic factors. The samples were obtained from 2017 till 2020, and all patients with initial diagnosis of cardiac myxoma with tumor size of at least 1 × 1 cm was included for the study. Size limitations were dictated by the need to forward part of the specimen for histopathological examination. Patients with smaller tumors than 1 × 1 cm or with papillary type of tumor unable to be subjected to histopathological examination due to too liquid/gelatinous structure were excluded from the study. Given the rarity of this tumor occurrence, it was assumed that specimens from at least five patients included for the study will be examined. The end of follow-up was marked by results from histopathological examination (2–3 weeks after surgery). This study was approved by the Bioethical Commission of the Jagiellonian University (approval number 1072.6120.123.2017) and an informed consent was obtained from each patient.

**Table 1:** Clinical characteristic of a study group

Patient ID	Sex	Age	Tumor localization	Tumor type	Tumor size (cm)	Metabolic disease	Cardiovascular disease	Diabetes
1	F	78	Left atrium	Solid	3 × 3	Hyperlipidemia	Hypertension, Ischemic heart disease, patent foramen ovale	–
2	M	57	Left atrium	Papillary	8 × 3	Extreme obesity	Paroxysmal atrial fibrillation, hypertension	–
3	F	59	Left atrium	Papillary	6 × 5	–	Hypertension, ischemic heart disease	Type II
4	F	84	Left atrium	Solid	3.5 × 2.5	Gout, hypercholesterolemia	Hypertension, paroxysmal atrial fibrillation, varicose veins, ischemic heart disease	-
5	F	52	Left atrium	Solid	3 × 2	–	–	–
6	M	64	Left atrium	Solid	2 × 2	Hypercholesterolemia chronic renal failure	Fixed atrial fibrillation, peripheral atherosclerosis, congestive heart failure	–

### Tissue sample preparation

During the surgery, the extracted tumor was aseptically sectioned into two pieces. While one piece was sent for the histopathology examination, the other one was placed in a sterile plastic container filled with Dulbecco's Modified Eagle's medium (DMEM) cell culture medium, high glucose (Cat. No. 61965026, Gibco®, Paisley, UK) with 10% fetal bovine serum (Heat Inactivated, Brazil Origin, Cat. No. 10500064, Gibco®, Paisley, UK), penicillin (10.000 U/mL), streptomycin (10.000 µg/mL) (Cat. No. 15140122 Gibco®, Paisley, UK), and amphotericin B (25 µg/mL) (Cat. No. 15290026, Gibco®, Paisley, UK). It was transported to the laboratory for the positron annihilation lifetime spectroscopy (PALS) examination within 4 h of extraction. Both myxoma and adipose tissues were sectioned into two pieces. Each PALS-measured sample consisted of two parts with the ^22^Na source in between (see Fig. 4a). After PALS measurement tissues were fixed in 4% formaldehyde in phosphate-buffered saline (PBS) for micro-CT imagining.

### Histopathology examination

We conducted histopathological examinations for all extracted tumors. Robust samples were fixed in 4% formaldehyde in phosphate-buffered saline (PBS), embedded in paraffin. The routine histopathology procedure was performed with H&E staining by a pathologist Dr. L. Rudnicka-Sosin MD and confirmed the primary diagnosis of cardiac myxoma for each of studied patients. The examination revealed elongated, round, or stellate single cells and tenuous cords, typically infiltrated by lymphocytes and macrophages, and surrounded by myxoid stroma.

### Cell culture

**Fig. 2:**
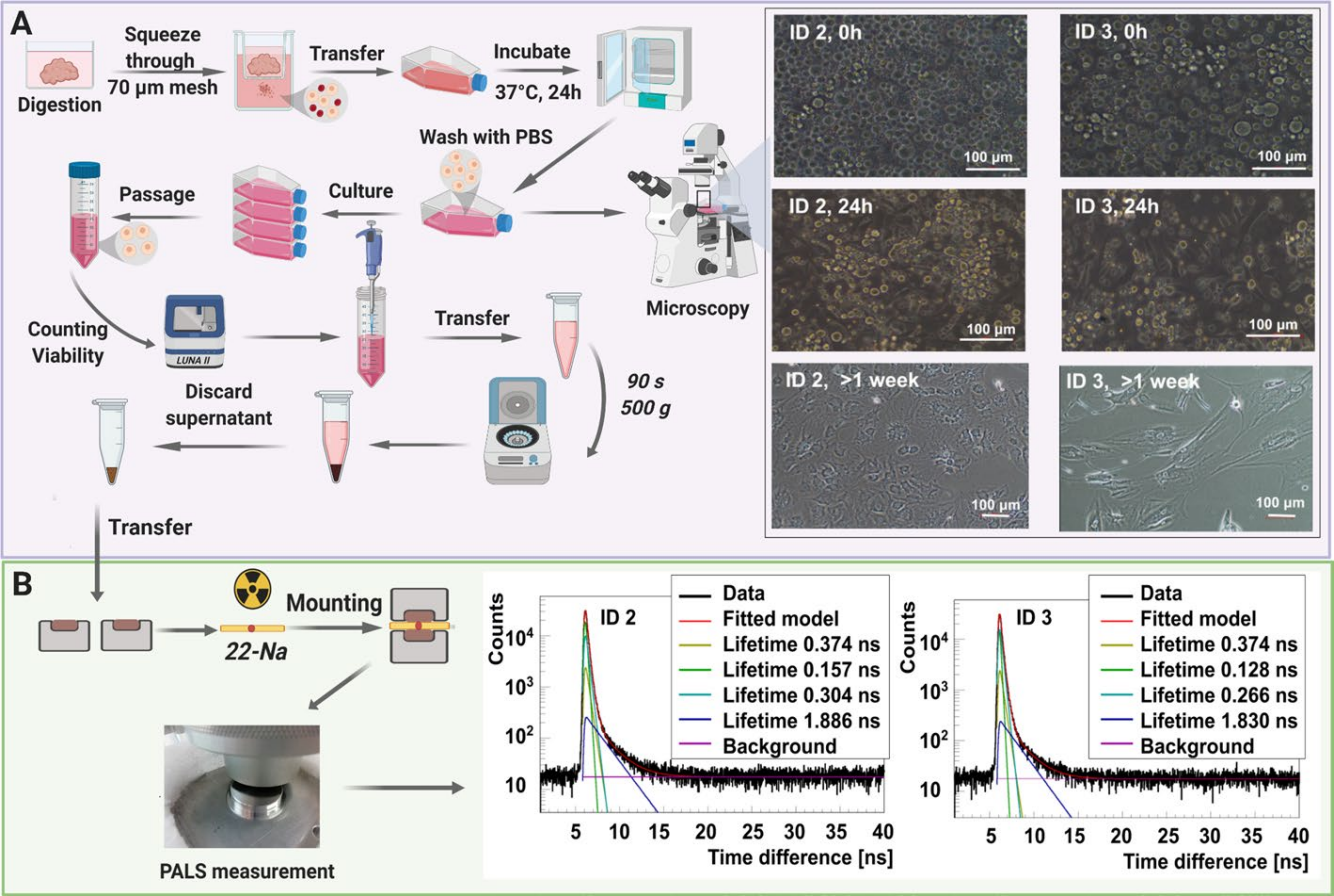
Myxoma cell culture isolated from the tumor of patient ID 2 and ID 3. **a** (Left) The workflow of myxoma cell culture isolation. Cells have been isolated by tissue digestion and cultured to obtain the highest possible number of cells for the experiment. A micrograph presenting the primary culture of cells isolated from cardiac myxoma, upon seeding, and 24 h later with erythrocyte contamination. The latter has been washed out during the primary culture. Secondary culture after the first passage has been established after 1 week. A scale bar of 100 μm. **b** (Left) The workflow of PALS measurement. The centrifuged cells have been placed in both parts of the aluminum chamber with a radioactive source (red dot) encapsulated between them. The chamber has been mounted between detectors in the temperature-controlled aluminum holder. The measurements have been taken at 37 °C. (Right) Positronium lifetime spectra with fitted components for the myxoma cell culture isolated from the given patient. The dark yellow, green, turquoise, and blue lines denote direct annihilation in the source material, p-Ps annihilation component, direct annihilation in the sample, and o-Ps annihilation component, respectively. The spectra are shifted by the offset coming from the detection system configuration (~ 5 ns). The patient ID annotations are described in Table 1

We derived myxoma cell cultures from two patients. The tissues were aseptically dissected with scalpel. We incubated the portion of tissue designated for cell isolation in a petri dish for 48 h. It comprised DMEM medium, high glucose (Cat. No. 61965026 Gibco™ Paisley, UK) with 10% fetal bovine serum (Cat. No. 10500064 Gibco™ Paisley, UK), penicillin (100 U/mL), streptomycin (100 µg/mL) (Cat. No. 15140122 Gibco™), amphotericin B (0.25 µg/mL) (Cat. No. 15290026 Gibco™), L-Glutamine (2 M) (Cat. No. 25030081 Gibco™ Paisley, UK), and collagenase II (200 U/mL) (Cat. No. 17101015 Gibco™, Paisley, UK). Following incubation, the tissue with the medium was squeezed through a 70 nm nylon mesh to isolate cells from the extracellular matrix. It was centrifuged at 260 g for 10 min, seeded on a T75 cm^2^ cell culture dish, and cultured at 37 °C and 5% CO2 atmosphere (Fig. 2a). Myxoma cells started attaching to the bottom of the dish after 24 h. We regularly washed the cells with PBS w/o Ca^2+^, Mg^2+^ (Cat. No. 10010015 Gibco™ Paisley, UK) during the first week of culture. This helped us wash out the erythrocytes. The cells were then incubated with 0.25% Trypsin-ethylenediamine tetra-acetic acid (Cat. No. 25200072 Gibco™ Paisley, UK) for 10 min, and removed from the flask with a cell scraper. We then centrifuged the cells in 260 g for 10 min. Following a spin, the cells were counted with Trypan Blue dye by an Automatic Cell Counter LUNA II, and seeded to the flasks at an appropriate density. Myxoma cells grow slowly and are usually seeded at a density of 2.5 × 10^5^ cell/cm^2^. They reach 80% confluence after 50 days. We prepared the cell samples for PALS measurement by passaging in a standard way, followed by counting the cells and suspending the pellet in a medium. It was later centrifuged in 500 g for 90 s. The supernatant was discarded thoroughly, and the cell pellet was transferred to a measurement chamber with a spatula. We determined the viability before and after each measurement to confirm that the setup conditions and cell handling were appropriate for the culture, and the high percentage of dead cells did not influence the results.

### Microscopy imaging

We regularly checked the cell morphology by means of a Nikon Eclipse TS100 inverted optical microscope. The photographs were captured with a Nikon DS-Fi1c camera and are presented in Fig. 2a. We conducted confocal microscopy studies for the cardiac myxoma cell culture on Zeiss Axio Observer Z.1 with an LSM 710 confocal module with Alexa FluorTM 647 Phalloidin (Cat. No. S32357 Invitrogen TM Paisley, UK) and the DAPI-stained (Cat. No. 62248 PierceTM Paisley, UK) for F-Actin and nucleus, respectively. VE-cadherin (BV9) Antibody (Cat. No. sc-52751, Santa Cruz Biotechnology, Inc.’s, US) and goat anti-mouse IgG-FIT secondary antibody (Cat. No. sc-2010, Santa Cruz Biotechnology, Inc.’s, US) with fluorescein isothiocyanate were used for the immunofluorescence staining. The images were processed with ZEN lite software.

### Positron annihilation lifetime spectroscopy

The positron annihilation lifetime spectrometer constituted two H3378-51 Hammamatsu photomultipliers, equipped with BaF_2_ cylindrical scintillators, with a diameter and height of 38 mm and 25 mm, respectively (see Fig. 4b). It had been manufactured by Scionix. Detectors were powered by CAEN SY4527 high voltage power supply. Signals from the photomultipliers were attenuated by 10 dB (indicated as A) and delivered to LeCroy 608C constant fraction discriminator (D). Different thresholds were applied to the signals from diverse detectors in the latter. Considering the varying thresholds set on the discriminator, while one detector was considered as ‘START’ (registers 1274 keV gamma quanta), the other one was considered as ‘STOP’ (registers 511 keV gamma quanta). We set the coincidence time window to 110 ns on the LeCroy 622 coincidence module. The data were acquired by a digitized DRS4 evaluation board. We used radioactive ^22^Na source with an activity of 1 MBq, sealed between 6 µm thick Kapton foils for all measurements. The samples were measured in an aluminum chamber with a source sandwiched between them. They were placed in a temperature-controlled holder. While the tissue samples were measured at room temperature, the cell culture samples were measured at 37 °C.

### Micro-CT

We used Lugol staining solution to enhance the X-ray attenuation within the samples [41]. The samples were fixed with 4% formaldehyde and placed in an individual dish containing 50 mL Lugol solution (I_3_K) (Cat. No. 62650 Sigma-Aldrich). They were stored for 5 days (4 °C). Before scanning, each sample was washed with saline solution and placed in individually designed 3-D printed sample holder to prevent motion during the scanning. We conducted the micro-CT investigation with Bruker SkyScan 1172 (Kontich, Belgium) scanner. The X-ray energy was set to 80 keV. No physical filter had been used. We captured the images with a pixel size of 8.97 µm. Each projection image was averaged out of eight frames to enhance the signal-to-noise ratio. Image reconstruction was performed using NRecon version 1.7.3.1 software by Bruker Micro-CT (Kontich, Belgium). The cross-sectional images were processed and analyzed using CTAnalyser 1.20.3.0 software by Bruker Micro-CT (Kontich, Belgium). The 3-D volume rendered models were prepared using CTVox 3.3.0 software by Bruker Micro-CT (Kontich, Belgium).

### Statistical analyses

The results of PALS are presented as the mean o-Ps lifetime and intensity. They were analyzed by the PALS Avalanche program, developed by the Jagiellonian-PET collaboration [42, 43]. Uncertainties of each measured point are a statistical error of the fitted function. The viability test and cell count were analyzed by means of the LUNA-II™ Automated Cell Counter (Logos Biosystems).

## Results

### CM tumors obtained from symptomatic patients were localized in a left atrium and they were characterized by different morphology

**Fig. 3:**
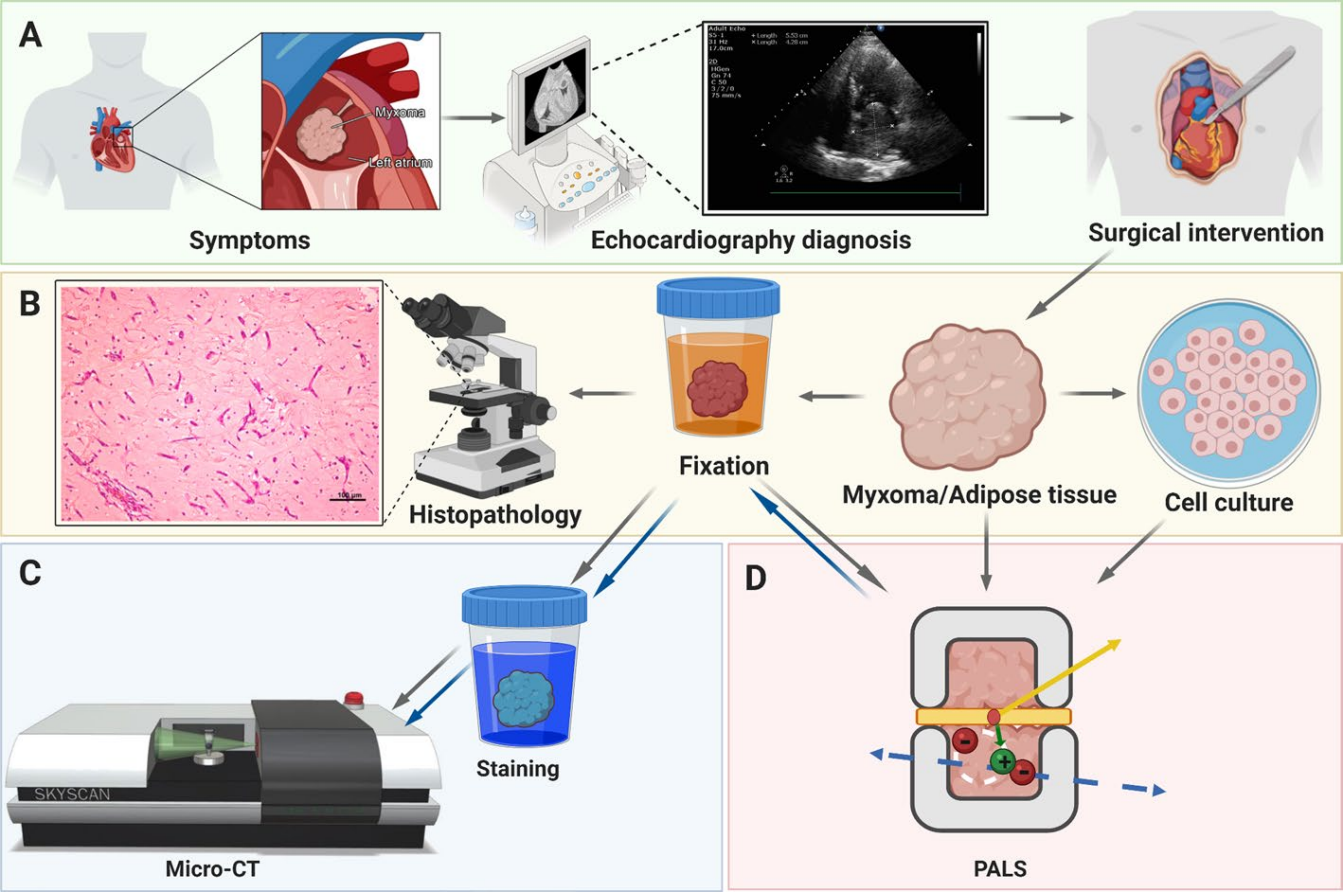
Cardiac myxoma experiment workflow. **a** The clinical examination of a symptomatic patient by transthoracic echocardiography and surgical excision of cardiac myxoma (CM). **b** Sample preparation and examination of myxoma, adipose tissues and CM cells: one piece of CM was fixed for histopathology examination (H&E), while other piece of CM and adipose tissue samples have been used for studying their positronium properties and later fixed for the X-ray imaging (micro-CT). CM cell culture has been derived from the part of tissue designated for PALS experiment before fixation. **c** Micro-CT imaging: staining in Lugol solution for 5 days and micro-CT scanning; gray and blue arrows represent a workflow for two different scanning runs: before and after positronium measurement (PALS). **d** A chamber for ortho-Positronium lifetime measurement comprising CM or adipose sample with the ^**22**^Na radionuclide (red dot) emits (green arrow) a positron (+) and a prompt photon (yellow arrow). Positron and electron from a sample create a positronium atom (bound state of electron and positron), indicated pictorially by a white dotted circle. An example annihilation of positronium into two photons (blue arrows). Figure 4 contains a detailed description of positronium lifetime measurement

We examined six patients, comprising four women. Their age ranged between 52 and 84 years. Moreover, they had multiple comorbidities, including metabolic and cardiovascular (Table 1) conditions. For each patient, we conducted a screening interview and laboratory test (blood counts, metabolic panel, and urine test), and diagnosed them based on the transthoracic TTE examination. The patients were symptomatic and had TTE-confirmed cardiac masses, localized in a left atrium. This necessitated the cardiac surgery (Fig. 3a). We obtained perioperative specimens of CM and mediastinal adipose tissues from the operating theater (John Paul II Hospital, Kraków). Half of each sample was fixed and designated for a histopathological analysis (Fig. 3b). In contrast, the other half was used for studying their positronium properties using PALS both in tissues and cell culture (Fig. 3b, d) and micro-CT examination (Fig. 3c).

**Fig. 4:**
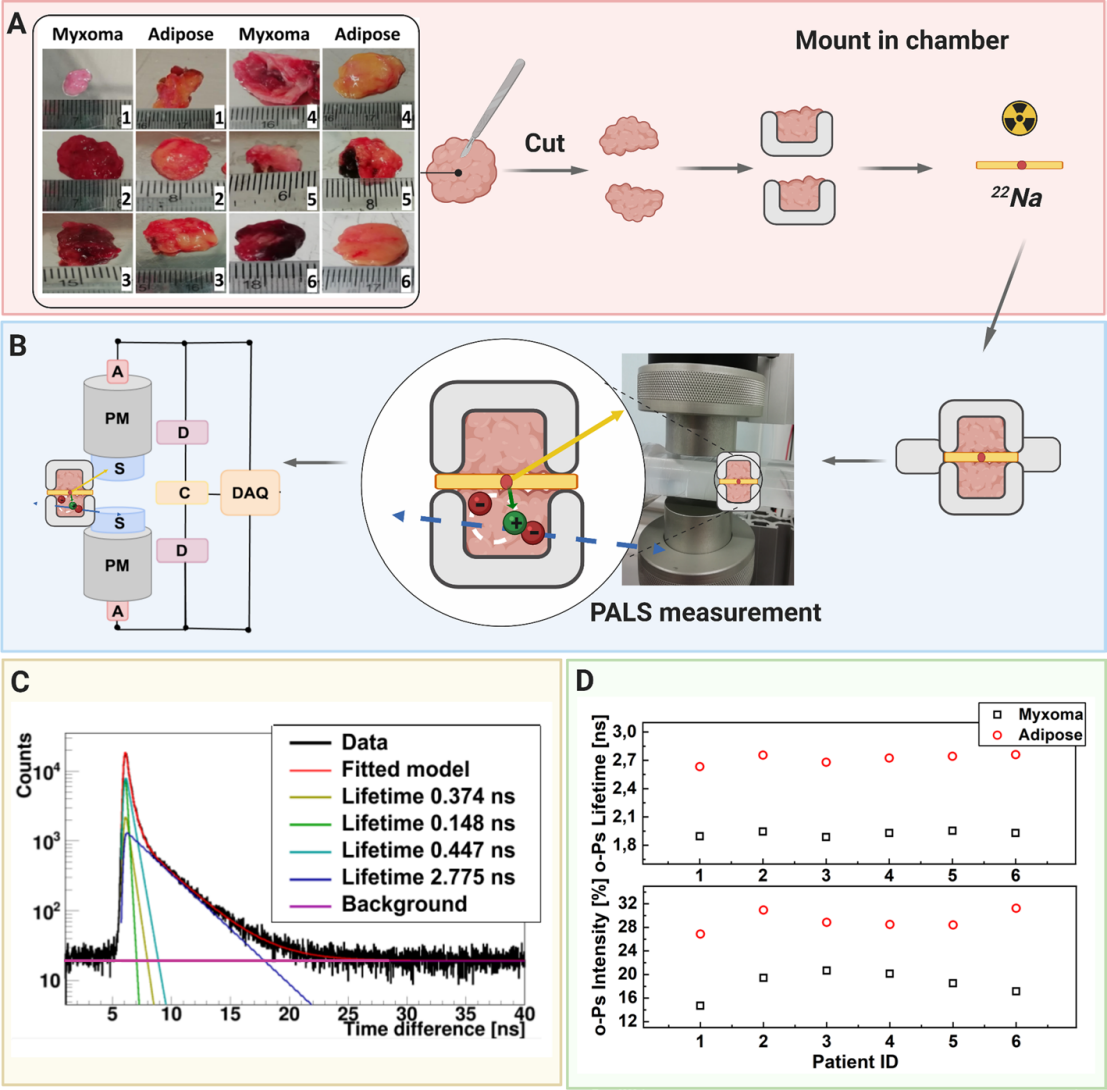
A scheme showing positronium lifetime measurements in cardiac myxoma and adipose tissues. **a** Photographs of non-fixed cardiac myxoma (CM) and adipose tissue samples. The numbers indicate patient ID (Table 1). Each sectioned tissue has been cut in halves with a ^22^Na radionuclide placed between and inserted into the aluminum measurement chamber. **b** The left part of the panel depicts the scheme of the detection system: scintillators (S), photomultipliers (PM), attenuators (A), discriminators (D), coincidence units (C), digitizer, and a data acquisition system (DAQ). The photograph displays a part of the system together with the plastic rod localized between the scintillators. The superimposed scheme indicates an aluminum chamber inserted inside the rod. ^22^Na (red dot) emits (green arrow) positron (+), which annihilates (predominantly into two photons indicated in blue) with electrons (−) in the tissue. Following the positron emission, ^22^Na changes into an excited nucleus of ^22^Ne, which de-excites almost instantly by the emission of the de-excitation photon (indicated in yellow). The PALS detection system, enables the measurement of the positronium lifetime by registering the time of emission of the de-excitation photon (corresponding to the time of positronium formation) and the time of creating the annihilation photons (corresponding to the time of the positronium decay). **c** For each sample, 1 × 10^6^ coincidences between annihilation and deexcitation *gamma quanta* have been registered, resulting in the lifetime spectrum (example for the adipose tissue—patient ID 2). The analysis enables the extraction of the mean lifetime and intensities of para-Positronium (green line) and ortho-Positronium (blue line) atoms trapped in the intramolecular voids [42]. The dark yellow, turquoise, and purple lines denote direct annihilation in the source material, direct annihilation in the sample, and the background because of the accidental coincidences, respectively. The spectra are shifted by the delay coming from detection system configuration (~ 5 ns). **d** Results of the mean ortho-Positronium (o-Ps) lifetime (upper) and intensity (lower panel) for CM (black squares) and mediastinal adipose (red circle) tissues

**Fig. 5:**
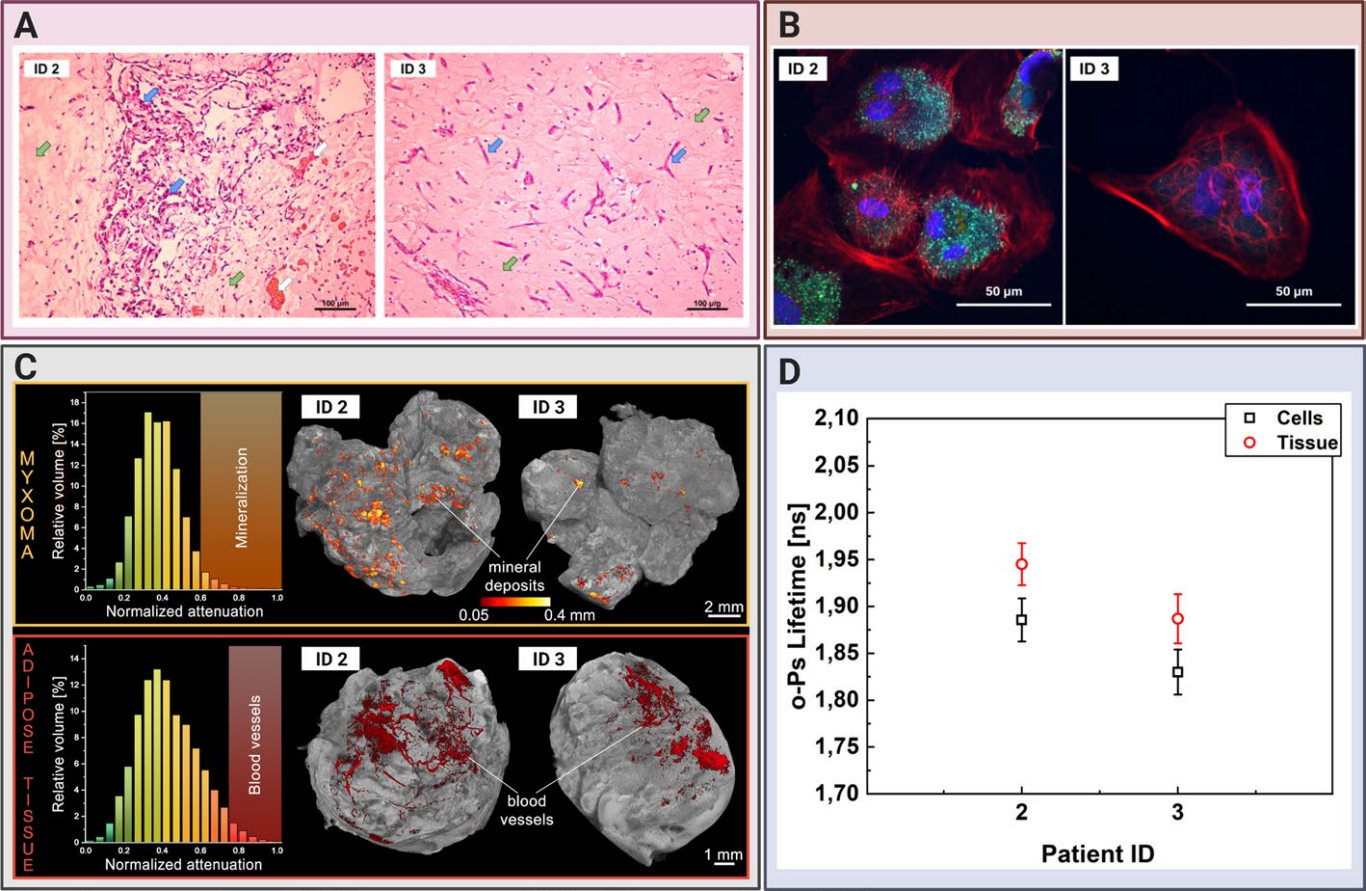
Comparing cardiac myxoma (CM) tissues and the isolated cell line. **a** Micrograph showing exemplary histopathology findings of cardiac myxoma (CM), for patient ID 2 and ID 3 in H&E staining. CM cells stained in purple (blue arrow) can have stellate (ID 3) or globular (ID 2) shape. The red/orange structures (white arrow) correspond to the blood vessels with erythrocytes. The surrounding myxoid matrix is stained in pink (green arrow). **b** Confocal microscopy image with the CM cells stained for F-Actin (red), nucleus (blue), and VE-cadherin (green). The scale bar is 50 μm. **c** Micro-computed tomography results for CM (upper row) and adipose tissues (lower row). Histograms on the left side present normalized X-ray attenuation within the sample: (i) the mineral deposits range from 0.6 to 1.0 in the CM samples; (ii) the blood vessels range from 0.75 to 1.0 in the adipose tissue samples. These attenuation ranges have been binarized to extract the mineral deposits and blood vessels for further analysis and visualization. The right side contains volume-rendered 3-D models of the two most representative samples, namely CM and adipose tissues. The internal mineral deposits have been highlighted in the CM model. Its diameter has been color-coded using a heat map. The blood vessels have been colored red in the adipose tissue. **d** Results of the mean ortho-Positronium (o-Ps) lifetime for CM tissue (red circles), isolated from the same patient myxoma cell line (black squares). Patients ID annotations are described in Table 1

Perioperative examinations and routine histopathology staining confirmed the primary diagnosis of CM. All cardiac tumors were pedunculated. They were diverse in size and structure. While four tumors were solid, two were of papillary type (see Table 1). In histopathology, we observed a typical hematoxylin and eosin (H&E) picture of a myxoma tumor with purple stellated or globularly shaped CM cells. Moreover, the orange-reddish structures represented the blood vessels with erythrocytes and the surrounding myxoid matrix was stained in pink (Fig. 5a). Mediastinal adipose tissue, also known as pericardial adipose tissue, was studied as a comparison to CM. It consists of cells called adipocytes, responsible for energy storage. White adipocytes, which can mostly be found in pericardial adipose tissue, are built as a single fat droplet, surrounded by cytoplasm with nucleus moved to peripheral side of the cell [44]. They are mainly composed of triglycerides, and cholesteryl ester.

Portions of samples used for the positronium investigations were sectioned and irradiated with positrons. We measured the positronium lifetime to compare its value in normal and tumor tissues. In addition, we derived the CM cell culture from two patients to observe the possible difference between the cellular and tissue specimens. Moreover, we conducted X-ray micro-CT imaging on the CM samples for the aforementioned patients to determine the structural heterogeneity of their tumors.

### The mean lifetime of o-Ps atoms in CM is shorter than in adipose tissues

Samples of CM and mediastinal adipose tissue obtained during the surgery were placed in a sterile plastic container, with a cell culture medium supplemented with 10% fetal bovine serum, and antibiotics. We maintained them for the in vitro PALS measurement (Fig. 4a) in the detector system. Each tissue sample was divided into two pieces and interlaced with a radioactive ^22^Na source in Kapton foil. The ^22^Na radioisotope emits positrons, which may penetrate the tissue up to about 1 mm [38], and annihilate with an electron from the biomolecule present in the tissue. The annihilation may proceed directly (e^+^e^−^ → photons) or via formation of positronium atom (e^+^e^−^ → positronium → photons). Positronium in 25% of cases is produced as short-lived (125 ps) para-positronium (p-Ps) and in 75% cases as long-lived (142 ns) ortho-Positronium (o-Ps). The measurements were taken at room temperature in an aluminum chamber (Fig. 4b) for 1 h to collect 1 × 10^6^ coincidences between annihilation and deexcitation gamma quanta per sample. Figure 4c presents the exemplary positronium lifetime spectrum obtained for the adipose tissues from the patient ID2. The superimposed lines indicate the distribution of components resulting from p-Ps (green), annihilation in the source material (dark yellow), free positron annihilation (turquoise), o-Ps (blue), and accidental coincidence background (purple). Each component is expressed as

$$I*e^{{\left( { - \frac{t}{\tau }} \right)}} ,$$

where I and τ denote the intensity and mean lifetime, respectively.

Figure 4d depicts the mean o-Ps lifetime obtained for the adipose (red circle) and CM (black squares) tissues. For all patients, we observed significant difference between the CM and normal adipose tissue both in o-Ps mean lifetime and intensity. While the averaged mean lifetime and intensity values for CM were 1.92(02) ns and 18.4(2.0)%, respectively, that for the adipose tissue were 2.72(05) ns and 29.1(1.5)%, respectively. The difference in o-Ps mean lifetime and intensity between CM and adipose tissue was 0.8 ns and 10.7%, respectively. In contrast, variations between the patients were around 0.03 ns and 4% in lifetime and intensity, respectively. Thus, there were significant differences in the o-Ps mean lifetime and intensity between normal and tumor tissues. Nonetheless, differences in the o-Ps lifetime and intensity originating from tissue heterogeneity or diversity in the study group did not influence the results.

### Micro-CT imaging and histopathology of CM revealed microscopic differences not influencing the mean o-Ps lifetime

Cardiac CT is commonly used as an additional noninvasive diagnostic modality in cardiovascular diseases, particularly for imaging the aorta and coronary arteries, visualizing the cardiac valves and heart anatomy, and for detecting intravascular and valvular calcifications [45]. We used micro-CT (enhanced with an iodine contrast agent) as a complementary technique to reveal the structure and composition of CM and adipose tissues. An analysis of the X-ray attenuation distribution within the samples from two representative patients revealed that high attenuation values corresponded with tissue mineralization in CM (normalized attenuation 0.6–1.0). A sample obtained from the patient ID2 had greater calcification spots, thus showing that CM tissues were not homogenous in structure. This in turn had been confirmed by the histopathology examination. The aforementioned patient had globular-shaped CM cells and vascularization spots (Fig. 5a). In cell culture, the CM cells isolated from the patient ID2 had a typical mesenchymal morphology and expressed the endothelial binding protein: vascular endothelial (VE)-cadherin [33]. In contrast, the CM sample from the patient ID3 had relatively fewer calcification spots. Moreover, the cells appeared to be stellate-like in histopathology. CM cells from this patient did not show VE-expression in cell culture (Fig. 5b). In addition, vascularization in the adipose tissues was represented and visualized for both patients showing different attenuation values than for calcification (normalized attenuation 0.75–1.0) (Fig. 5c). We did not detect blood vessels in CM. Similarly, there were no mineral deposits in the adipose tissues. There is a significant diversity in the spatial distribution of highly attenuating regions in the samples, particularly in the dispersion of mineral deposits.

In contrast to the calcification deposits revealed by micro-CT, microvessels cannot be visualized in CM with the above-mentioned technique. This necessitates a higher resolution approach. The use of positronium imaging as a complementary method to CT or MRI can distinguish the histological changes and structural differences on a nanometer level.

### Comparing the CM tissue and the isolated cell line reveals similar o-Ps lifetime characteristics

We established the myxoma cell culture from tissues operated from two patients, namely ID2 and ID3. This helped us compare the mean o-Ps lifetime in the CM cells and CM tissues, which comprise a milieu of extracellular matrix and blood vessels, in addition to cells. The Methods Section in Fig. 2a outlines the workflow of CM cell isolation, culture, and sample preparation for the positronium lifetime measurement. Furthermore, it also presents micrographs of cell cultures upon seeding (0 h), 24 h later, and the secondary culture. We observed the floating CM cells and the number of erythrocytes while seeding. The CM cells attached to the surface of the culture dish surface 24 h later. The remaining debris and erythrocytes were washed out. After a week following the isolation, the CM cells developed a mesenchymal-like shape having multiple nuclei, mostly around 2–3 per cell in the secondary culture. The myxoma cells had a diameter of roughly 40–50 µm (Fig. 5b).

For the positronium lifetime analysis, the CM cells were harvested from the cell culture, sedimented to obtain roughly 20 million cells (Fig. 2a), and placed in an aluminum chamber at 37 °C for 1 h (Fig. 2b). We determined the cell viability before and after the measurement and obtained a difference of 10.0(2.3)%. The exemplary positron lifetime spectra for the measured samples are presented in the Methods Section in Fig. 2b.

The determined mean o-Ps lifetime for the cultured CM cells (red circles) from both patients (ID2 and ID3) was lower than those for the raw CM tissues (black squares): 1.89(3) ns vs 1.95(1) ns and 1.83(3) ns vs. 1.89(3) ns, respectively (Fig. 5d). These results are present within 2 standard deviations. Despite removing the surrounding tissue matrix from the myxoma sample, such as extracellular proteins and vessels, the tendency to obtain a higher signal for the patient ID2 was preserved. CM cells from the patient ID2 expressed greater adhesion proteins (for example, VE-cadherins), which may be related with different molecular structures and metabolism, and influence the o-Ps lifetime (Fig. 5b).

## Discussion

Methods used to confirm a CM diagnosis varied in past decades. Earlier, patients were diagnosed by cardiac catheterization. However, TTE is performed routinely in recent times, with MRI or CT scan imaging being used as a complementary diagnostic modality [27–29, 46, 47]. TTE can be extended by a transesophageal approach that facilitates an intraoperative cardiac investigation to confirm tumor localization [29, 46].

In practice, echocardiography can usually distinguish between the three intracardiac masses, namely tumor, thrombus, and vegetation, with an accuracy 80% [47, 48]. A presumptive TTE diagnosis can be made for primary cardiac neoplasm, including CM, for some distinctive features [23]. Therefore, MRI and CT are constantly needed to improve the definitive diagnosis. Nonetheless, these techniques provide limited tissue characterization.

The recent advent of high sensitivity total-body PET [3, 5, 6, 9] and the invention of positronium imaging offer new perspectives for improved diagnostic and prognostic assessment, based on cell and its milieu alterations at the molecular level [1, 4, 13–16, 49]. Positronium is copiously produced in the intramolecular spaces during routine PET imaging. It can be effectively used as a diagnostic parameter, complementary to the standardized uptake value image [1, 4, 39]. We demonstrated for the first-time substantial differences in the positronium mean lifetime and the production probability for cardiac neoplasm (myxoma), compared to normal mediastinal adipose tissues. Thus, our findings offer promising prospects for improving the diagnosis of heart diseases using positronium imaging. Notably, the determined differences of the mean positronium lifetime in CM and adipose tissues are at the level of more than ten standard deviations for each examined patient. However, the mean o-Ps lifetime values in CM were constant within two standard deviations in the study group of patients.

PET is currently used as a standard in cardiology for myocardial perfusion evaluation [18, 34–37]. Therefore, the new functionality of PET devices that enables positronium imaging, simultaneously with standard SUV imaging, shall constitute a natural enhancement of the diagnosis by preserving the current PET imaging protocol.

We used sodium ^22^Na isotope as an emitter of positrons (e +), considering the role of sodium fluoride in cardiovascular imaging [34, 37]. An example of a radiopharmaceutical applicable for the positronium imaging of cardiovascular system is the Food and Drug Administration–approved ^82^Rb-Chloride. This in turn would facilitate assessing the perfusion of the cardiovascular system [3, 50]. Similar to ^22^Na, ^82^Rb isotope also emits a prompt photon, in addition to a positron. Sitarz et al. have reported on prompt photon positron emitters that look promising for PET imaging [51]. Concurrent application of the β + γ emitter with a novel radiotracer, e.g., the ligand of serine protease fibroblast activation protein (FAP), which is a biomarker of activated (myo)fibroblasts, will give a new advantage in assessment of post-myocardial infarction remodeling [52]. Moreover, examples of tracers proposed for PET imaging with the aforementioned isotopes are summarized in reference [3, 53]. Observed differences in o-Ps lifetime between both adipose and myxoma tissue and myxoma cell cultures vs tissues can originate from different intramolecular void structure and free radicals and oxygen concentration. It was shown by studies of various biological samples that each of this factor is contributing to o-Ps lifetime values [54–60]. Detailed understanding of the relative contribution of various electron–positron annihilation mechanisms requires further systematic investigations. However, as discussed in the recent review [61], in the biological sample we may approximately state that the pick-off process rate (~ 512.8 μs^−1^ in water) is much larger than conversion and other reactions rate (~ 27 μs^−1^ in O_2_ saturated water) [61]. The difference of the oxygen concentration between healthy and cancer tissues are ranging between 20 μmol/L and 90 μmol/L [62]. Based on measurements reported in references [16, 40] it was shown that such differences in the oxygen concentration may cause changes of the o-Ps lifetime at the order of 5 ps only [62]. Therefore, we may conclude that the differences in o-Ps lifetime between myxoma and adipose tissues reported in this work, being as large as ~ 800 ps, are predominantly due to the differences in the pick-off process rates. Hence, they are due to the structural differences at the molecular level [61]. Such large differences indicate that o-Ps lifetime may become useful in clinics as a tissue pathology indicator. The perspectives for translation of positronium imaging into clinics were recently discussed in reference [63]. It was advocated that 40 times higher sensitivity of the new generation PET systems [3, 64, 65] combined with multi-photon acquisition technique [1, 2], and the newly developed iterative methods for positronium imaging [66–68] enables reconstruction of positronium images with the spatial resolution of about 4 mm and o-Ps lifetime resolution of 20 ps [1], making the positronium imaging applicable in clinics.

## Conclusion

In summary our results demonstrated the usefulness of positronium (its lifetime and intensity) to differentiate between pathologies, that is, benign cardiac tumors and adipose tissues. These data evaluate an o-Ps lifetime as a new biomarker for cardiovascular imaging, particularly for imaging intracardiac masses to reveal their structural composition. Structural heterogeneity is characteristic of CM tumors. However, we showed that the macroscopic (morphology) and microscopic (micro-CT) structural changes did not influence the value of the observed parameter (mean o-Ps lifetime), which reflected changes on a molecular (nanoscale) level.

## Data Availability

Data will be made available upon reasonable request.
